# Coffee as a Deodorizer

**Published:** 1869-03

**Authors:** 


Coffee as a Deodorizer.—Coffee is spoken of in high terms by foreign journals as a deodorizer for the neutralizing of foul odors that emanate from organic bodies in a state of decay, as it can be used to advantage where other disinfecting agents would be inadmissible. In cases where rats die in the spaces between the floors of dwellings, the intolerable odor arising therefrom can be effectually removed by placing a pound or two of fresh burnt and ground coffee between the floors. For the purification of a sick room it is incomparably superior to burning rags, as it has a beneficial chemical action on the atmosphere of the room, and gives, besides, an agreeable perfume.

